# Bannwarth Syndrome in a Patient With Early-Stage Breast Cancer: An Atypical Manifestation of Lyme Neuroborreliosis

**DOI:** 10.7759/cureus.103078

**Published:** 2026-02-06

**Authors:** Madison S Meyer, Miranda Goodson, Sarah R Eggert-Cichocki, Benjamin Frimodig, Hasan Sawan, Khalid Zakaria

**Affiliations:** 1 Department of Medicine, Wayne State University School of Medicine, Detroit, USA; 2 Internal Medicine, Henry Ford Health System, Novi, USA; 3 Department of Internal Medicine, Wayne State University School of Medicine, Detroit, USA; 4 Department of Psychiatry, Wayne State University School of Medicine, Detroit, USA

**Keywords:** bannwarth syndrome, breast cancer, invasive ductal carcinoma, lyme neuroborreliosis, lymphocytic meningoradiculitis, paraneoplastic neuropathy

## Abstract

Neurological symptoms in breast cancer patients can arise from metastasis, medication side effects, or underlying neurodegenerative conditions. When imaging is inconclusive, the more uncommon immune-mediated neuropathies should be considered. Guillain-Barré syndrome and Lyme neuroborreliosis are two immune-mediated conditions that share neurological findings. This case involves a 72-year-old Caucasian female who presented with early-stage invasive ductal carcinoma with neuroendocrine features and gradual bilateral upper extremity weakness and paresthesia. Surgery was uncomplicated, but in the subacute postoperative period, the patient developed progressive worsening of neck and scapular pain, left upper extremity weakness, right-hand paresthesia, and decreased right-hand dexterity. Cervical spine MRI and CT scans of the head and neck areas were insignificant. Leukocytosis, mild hyponatremia, and elevated C-reactive protein were found on laboratory workup. The initial differential diagnosis included paraneoplastic syndromes; however, given the inconclusive imaging, immune-mediated neuropathies, including Lyme neuroborreliosis, were prioritized. A lumbar puncture established the diagnosis of Lyme neuroborreliosis presenting as Bannwarth syndrome, also known as lymphocytic meningoradiculitis. Acute or subacute neurological decline in oncology patients should prompt consideration of paraneoplastic and infectious immune-mediated neurological disorders. Despite its rarity in the United States, Bannwarth syndrome should be considered as a paraneoplastic neuropathy mimic, especially with inflammatory cerebrospinal fluid findings.

## Introduction

Breast cancer is the most frequently diagnosed cancer in women worldwide [1]. Neurological manifestations often accompany breast cancer due to metastatic spread, cancer treatment-related neurotoxicity, or an unrelated degenerative disease [2]. Paraneoplastic neurological syndromes (PNS) are uncommon in patients with breast cancer and are associated with high morbidity [3]. Paraneoplastic neurological disorders can present with a variety of neurological symptoms, and often demonstrate few, if any, abnormalities noted on neuroimaging [4,5]. Similar neurologic findings can be the result of other infectious diseases.

*Borrelia burgdorferi*, a spirochete infection, causes Lyme neuroborreliosis [6]. Early Lyme disease commonly presents with erythema migrans and nonspecific systemic symptoms such as fever, fatigue, headache, and myalgias [7]. Neurological manifestations from Lyme neuroborreliosis can occur rarely and can include radiculopathy, cranial neuropathies, or limb weakness [8]. Lyme neuroborreliosis with atypical presentations that lack the classic “Bull's-eye” rash, flu-like symptoms, and absence of known tick exposure history can have presentations mimicking paraneoplastic syndromes in addition to other immune-mediated neuropathies [9]. We present a case of a patient with newly diagnosed breast cancer alongside unexplained neurological symptoms, leading to the diagnosis of Lyme neuroborreliosis with atypical manifestations.

## Case presentation

**Figure 1:**
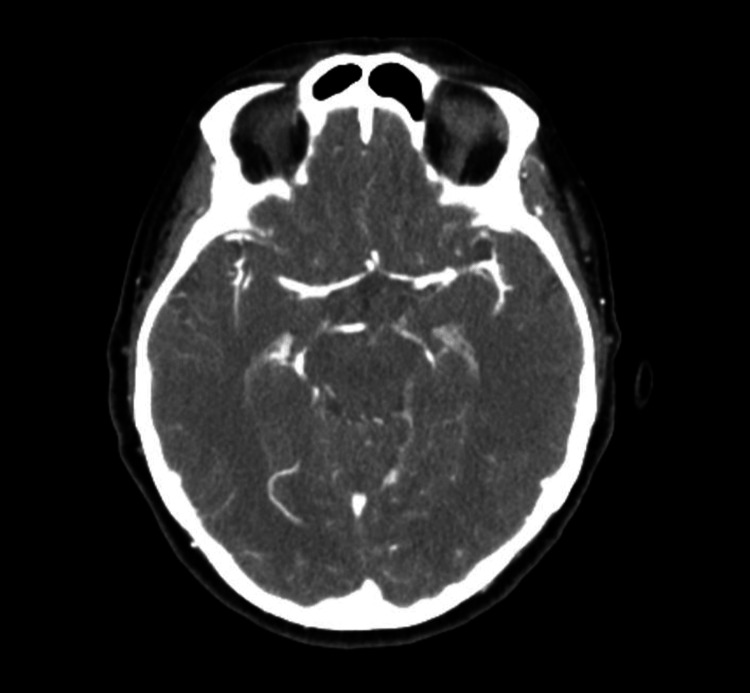
Axial CT angiography of the head demonstrating normal intracranial arterial opacification. The major anterior and posterior circulation vessels show no evidence of stenosis, occlusion, aneurysm, dissection, or other vascular abnormality. No acute intracranial hemorrhage or mass effect is identified.

**Figure 2:**
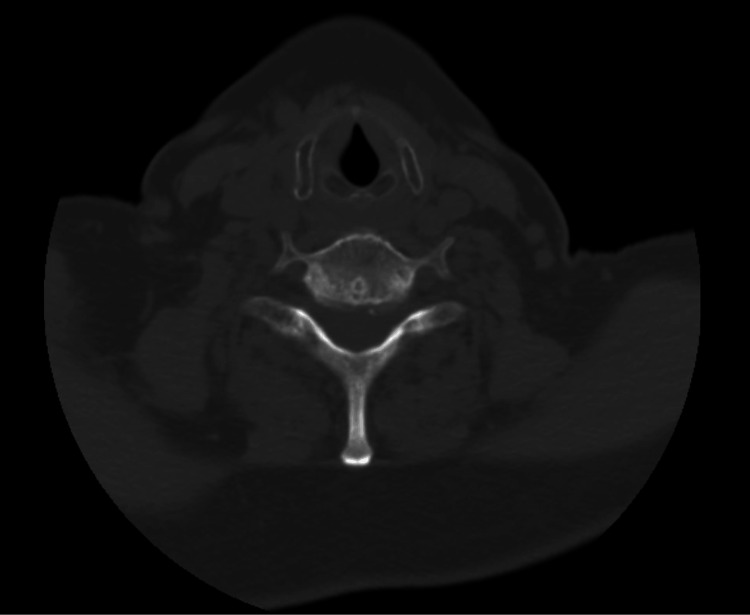
Axial non-contrast CT scan at the C5–C6 level demonstrating normal cervical spine anatomy. The vertebral bodies, spinal canal, and surrounding soft tissues appear unremarkable, with no evidence of fracture, malalignment, canal stenosis, or acute soft-tissue abnormality.

A 72-year-old Caucasian woman with a history of hypertension, hypothyroidism, obesity, and early-stage invasive ductal carcinoma with neuroendocrine features presented to the emergency room complaining of generalized neck and shoulder pain. At the time of admission, she had not undergone chemotherapy or radiation treatment. She was prescribed ketorolac, a lidocaine patch, and methocarbamol with minimal improvement. During admission, a computed tomography angiography (CTA) of the head and neck and a CT of the cervical spine and head without contrast were completed and were unremarkable (Figures 1-2). In conjunction with the imaging, unremarkable clinical exam, and labs, she was discharged home and returned three weeks later for a scheduled lumpectomy.

One day, following an uncomplicated right lumpectomy, the patient presented with gradually worsening upper extremity weakness. On examination, the patient was fully alert and oriented with intact cranial nerves II through XII. The left upper extremity demonstrated one out of five strength proximally with mild grip weakness; the right upper extremity had normal strength but had sensory changes in the second and third fingertips. She also experienced cervical and paraspinal tenderness, while the lower extremity demonstrated full strength bilaterally. Initially, radiculopathy was suspected.

**Figure 3:**
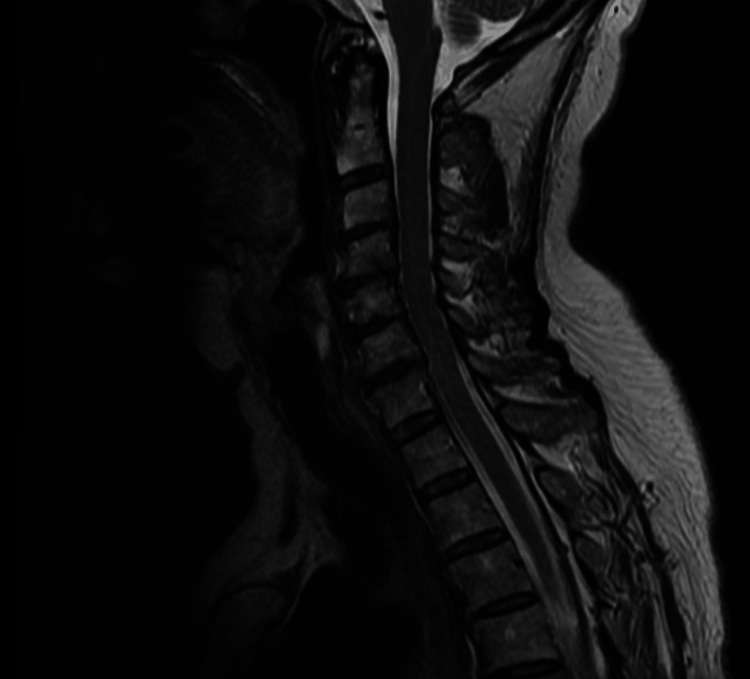
Sagittal view, T2-weighted MRI of the cervical spine, with no nerve root or leptomeningeal enhancement. Mild degenerative disc changes were present across C3-C7.

**Figure 4:**
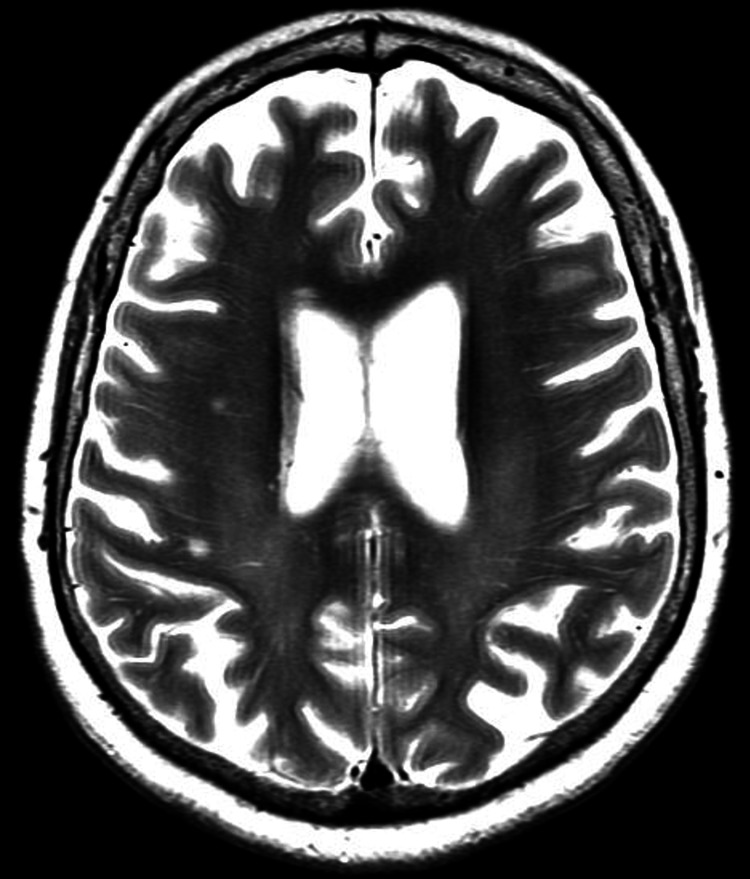
Axial view, T2-weighted MRI of the brain, with no noted parenchymal lesions or leptomeningeal enhancement.

The lab results showed an initial leukocytosis (12.01 K/mcL), hyponatremia (129 mmol/L), and elevated C-reactive protein (CRP) (13.8 mg/L) with an unremarkable erythrocyte sedimentation rate. The patient was admitted for observation and started on morphine, ketorolac, and dexamethasone, all of which provided minimal relief of symptoms. An MRI of the cervical spine and brain with and without contrast was ordered and showed mild degenerative changes at C3 through C7, with mild canal stenosis and cord abutment at C3-4 and C6-7, but no significant lesion or enhancement (Figures 3-4). Additional tests, including lumbar puncture with cerebrospinal fluid (CSF) culture with Gram stain, electrocardiogram (EKG), and a full polymerase chain reaction (PCR) viral panel, were all unremarkable.

Due to the progression of neurological symptoms despite standard imaging results and supportive treatment, a lumbar puncture was performed (Table 1). The detection of *Borrelia burgdorferi *antibodies in both the serum and CSF fluid with intrathecal production confirmed the diagnosis of Lyme neuroborreliosis. The patient was started on intravenous ceftriaxone 2 g every 12 hours for four weeks. Within one week of starting therapy, the patient reported her symptoms had steadily improved. Her home dose of gabapentin was increased to manage the radiculopathy.

**Table 1:** Cerebrospinal fluid confirming Lyme neuroborreliosis.

Test	Result	Reference range	Interpretation
Protein (mg/dL)	140.0 ↑	15.0-45.0	Markedly elevated
Glucose (mg/dL)	72	40-70	Normal
*B. burgdorferi* IgG Ab index	1.58 ↑	<0.14	Positive
*B. burgdorferi* IgM Ab index	>4.50 ↑	<0.07	Strongly Positive
*B. burgdorferi* PCR	Not detected		Negative
Culture	No growth		Negative
ACE (unit/L)	2.3	0.0-3.1	Normal

Retrospectively, the patient noted redness around the site of her breast neoplasm that resolved prior to admission, but assumed the rash was from lymphedema or cellulitis. It remained unclear if this represented an early manifestation of Borrelia burgdorferi infection. The distribution of this erythema did not clearly correspond to the dermatomes later involved in the patient’s radiculopathy, which limited definitive clinical correlation.

## Discussion

This case describes a patient with newly diagnosed invasive ductal carcinoma with confirmed Lyme neuroborreliosis that manifested as Bannwarth syndrome. Bannwarth syndrome (also known as lymphocytic meningoradiculitis) is a subtype of classic Lyme neuroborreliosis, as it presents with painful radiculoneuritis and lymphocytic pleocytosis in the CSF [10]. It is more common in Europe and uncommonly found in the United States [11]. This represents a rare and atypical finding, illustrating the concurrent manifestation of an uncommon infectious neuropathy alongside a common malignancy.

There were many diagnostic challenges, highlighted by the patient’s atypical presentations that mimicked PNS. Immune-mediated paraneoplastic neuropathy was considered the initial diagnosis due to the patient’s subacute onset of bilateral weakness in the upper extremity, radicular pain, and sensory deficits on physical exam [12].

Although lymphocytic meningoradiculitis can manifest on CNS imaging as nerve root and leptomeningeal enhancement on contrast-enhanced MRI, this was absent in the patient [13,14]. This absence is not uncommon and highlights the frequent dissociation between imaging results and the clinical phenotype of the disease [15].

A different diagnosis was prompted when lymphocytic pleocytosis and increased protein were found on CSF analysis. The patient’s serum and CSF tests were confirmed positive for *Borrelia burgdorferi *antibodies with intrathecal antibody production, which usually takes four to six weeks to become detectable [9]. This confirmed the diagnosis of neuroborreliosis.

In North America and Europe, Lyme disease is the most common tick-borne disease [16]. However, it is unusual for Lyme disease to present with neurological symptoms so early, as it usually manifests weeks to months following the initial infection [9]. While early Lyme neuroborreliosis usually presents with facial nerve palsy, lymphocytic meningitis, and painful radiculoneuritis, patients presenting without erythema migrans or a known tick exposure are often overlooked [17]. The absence of rash and outdoor exposure makes Lyme neuroborreliosis initially an unlikely diagnosis [18]. Later in the hospital course, the combination of neurological symptoms and CSF inflammatory findings led to targeted Lyme neuroborreliosis testing and diagnosis.

This case demonstrates why clinicians should keep infectious mimics in mind when assessing cancer patients who show new neurological symptoms on clinical exam with no metastatic lesions in neuroimaging. Malignancy can be associated with neurological deficits either due to tumor progression or paraneoplastic phenomena, which can delay the consideration of and treatment for an underlying, treatable infectious process, such as Lyme neuroborreliosis [2]. Furthermore, the presence of breast cancer complicated this patient’s diagnostic workup since immune-mediated paraneoplastic neuropathies have similar, if not identical, clinical presentations.

The diagnosis of Lyme neuroborreliosis led to the early use of intravenous ceftriaxone [19]. Eventually, this medical intervention allowed for a slow but meaningful neurological recovery. This highlights the reversibility of neurological deficits in patients with neuroborreliosis with early treatment [20]. As a result of this case, a higher value is placed on an early lumbar puncture with serologic testing in the workup of unexplained neurological decline, even when the pre-test probability of an infectious etiology is low. This case is a reminder that a broad and systematic approach to the diagnostic process can avoid curative opportunities in complex oncology patients.

## Conclusions

This case report describes a 72-year-old Caucasian female with invasive ductal carcinoma status post-lumpectomy who presented with gradually worsening weakness and was subsequently diagnosed with Lyme neuroborreliosis, particularly the Bannwarth syndrome type. This case underscores the importance of including Bannwarth syndrome in the differential diagnosis of subacute neurological deterioration, particularly in oncology patients with inflammatory cerebrospinal fluid findings and unrevealing neuroimaging, even in regions where the condition is less commonly reported. Especially in patients with cancer, neurological deterioration can stem from a variety of etiologies, not just their treatment or the cancer itself. Prompt and comprehensive consideration of paraneoplastic, immune-mediated, and infectious causes is essential to ensure accurate diagnosis and efficacious treatment that prevents permanent neurologic damage, ultimately leading to better functional results.
